# Human kidney clonal proliferation disclose lineage-restricted precursor characteristics

**DOI:** 10.1038/s41598-020-78366-3

**Published:** 2020-12-16

**Authors:** Osnat Cohen-Zontag, Rotem Gershon, Orit Harari-Steinberg, Itamar Kanter, Dorit Omer, Oren Pleniceanu, Gal Tam, Sarit Oriel, Herzel Ben-Hur, Guy Katz, Zohar Dotan, Tomer Kalisky, Benjamin Dekel, Naomi Pode-Shakked

**Affiliations:** 1 Pediatric Stem Cell Research Institute, Edmond and Lily Safra Children’s Hospital, Sheba Medical Center, Tel-Hashomer, Israel; 2 Department of Urology, Sheba Medical Center, Tel-Hashomer, Israel; 3 The Talpiot Medical Leadership Program, Sheba Medical Center, Tel-Hashomer, Israel; 4 Faculty of Engineering and Bar-Ilan Institute of Nanotechnology and Advanced Materials (BINA), Bar-Ilan University, Ramat Gan, Israel; 5 The Joseph Buchman Gynecology and Maternity Center, Sheba Medical Center, Tel-Hashomer, Israel; 6 Division of Pediatric Nephrology, Edmond and Lily Safra Children’s Hospital, Sheba Medical Center, Tel-Hashomer, Israel; 7 Sackler Faculty of Medicine, Tel-Aviv University, Tel-Aviv, Israel; 8 L.E.M. Laboratory of Early Detection, Nes Ziona, Israel; 9 Department of Obstetrics and Gynecology, Shamir Medical Center, Be’er Ya’akov, Israel

**Keywords:** Cell biology, Diseases, Medical research, Molecular medicine, Nephrology, Biological techniques, Bioinformatics, Biological models, Gene expression analysis, Sequencing

## Abstract

In-vivo single cell clonal analysis in the adult mouse kidney has previously shown lineage-restricted clonal proliferation within varying nephron segments as a mechanism responsible for cell replacement and local regeneration. To analyze ex-vivo clonal growth, we now preformed limiting dilution to generate genuine clonal cultures from one single human renal epithelial cell, which can give rise to up to 3.4 * 10^6^ cells, and analyzed their characteristics using transcriptomics. A comparison between clonal cultures revealed restriction to either proximal or distal kidney sub-lineages with distinct cellular and molecular characteristics; rapidly amplifying de-differentiated clones and a stably proliferating cuboidal epithelial-appearing clones, respectively. Furthermore, each showed distinct molecular features including cell-cycle, epithelial-mesenchymal transition, oxidative phosphorylation, BMP signaling pathway and cell surface markers. In addition, analysis of clonal versus bulk cultures show early clones to be more quiescent, with elevated expression of renal developmental genes and overall reduction in renal identity markers, but with an overlapping expression of nephron segment identifiers and multiple identity. Thus, ex-vivo clonal growth mimics the in-vivo situation displaying lineage-restricted precursor characteristics of mature renal cells. These data suggest that for reconstruction of varying renal lineages with human adult kidney based organoid technology and kidney regeneration ex-vivo, use of multiple heterogeneous precursors is warranted.

## Introduction

With end stage renal disease becoming a worldwide challenge and subsequently the need for kidney donors expanding, far exceeding their availability, there is a burning need for alternative regenerative approaches. For both tissue engineering and direct cellular interventions, utilization of cell sources harboring bonafide renal potential, e.g. renal lineage cells that function to generate or replace mature kidney cells, may prove beneficial^1^. For that to occur attempts at understanding renal cells growth are of importance.

Previously, we obtained a comprehensive view of in vivo mouse kidney epithelial cell dynamics at the single-cell level^2^. Using multicolored fluorescent fate-mapping mouse models to track the behavior of single epithelial cells and clonal units, we discovered that cell turn-over in the nephron relies on multiple foci of clonal proliferation of kidney-resident epithelial cells that are dispersed along the nephron and collecting system. Long-term in vivo analysis of clonal progeny showed each clone to contribute to a single renal epithelial lineage indicating that adult kidney growth was segmented. Thus, our study show widespread potential of renal parenchyma to act as lineage progenitors for local cell replenishment and regeneration^4^. Moreover, Wnt-responsive cells could preferentially generate larger clones but clonal proliferation was not exclusive to these cells^2^. Thus, clonal proliferation is a mechanism of cell replacement in the adult kidney and single cells can be induced to replace lost cells via clonal proliferation. Similarly, using lineage tracing in mice, LGR5 + cells were shown to mark clonal behavior of distal tubular epithelial cells in development^3^, while Schutgens et al. showed that cells expressing the *Tnfrsf19* (also known as *Troy*), clonally contribute to tubular structures during development and continue to give rise to segment-specific collecting duct cells in homeostasis of the adult kidney^4^.

Analyzing kidney repair following acute damage, intrinsic proximal tubule cell proliferation was shown to possess a major role in replacing the lost cell mass^5,6^. In addition, several evidence support epithelial dedifferentiation and proliferation to ensue kidney injury^7^. Recently, in vivo lineage tracing of proximal tubule KIM1 + cells (a marker specific for injured proximal tubule cells) early following acute kidney injury and clonal analysis, revealed clonal expansion and co-expression of KIM1, VIMENTIN, SOX9, and MKI67 in their derived clonal progeny, suggesting concomitant mesenchymal dedifferentiated and proliferative states of these clonal cells^8^.

In the adult human kidney enhanced in vitro clonal proliferation has been shown to occur in sorted cell fractions expressing markers such as CD133, CD24, ALDH1A1, NCAM1^8–11^. Nevertheless, our findings of wide CD133/CD24 expression in differentiated epithelial cells in the human fetal kidney^12,13^ as well as their broad expression (> 80%) in early passage bulk cultures of the human adult kidney suggest that these markers may signify proliferating mature tubular epithelial cells^14^.

Herein we developed a culture system which enables clonal proliferation and propagation of one single kidney epithelial cell derived from human adult kidneys and analyzed clonal behavior by transcriptomics. Our results show mature renal parenchymal cells clonally proliferate/propagate mostly as precursors restricted to sub-lineages. This includes distinct types of proximal and distal single-cell kidney derived clones harboring specific molecular characteristics that correspond to in vivo tubular cell traits and unveil possible molecular drivers and markers for human kidney clonal cell proliferation. Importantly, when compared to bulk counterparts’ early clones appear to be more quiescent (reduced Ki-67) and show a consistent pattern of renal marker reduction while preserving renal identity markers of several segments (multiple identity signature), concomitant with renal developmental gene activation.

## Materials and methods

### Ethics statement

This study was conducted according to the principles expressed in the Declaration of Helsinki. The study was approved by the Institutional Review Boards of Sheba Medical Center, Wolfson Hospital and Asaf Harofeh Medical Center. All procedures were carried out following signed informed consents.

All pregnant women involved in the study provided written informed consent for the collection of samples from their aborted fetuses and subsequent analysis.

### Establishment of primary cultures from the human kidney

Human adult kidney samples were recovered from healthy margins of renal cell carcinoma (RCC) tumors, resected from partial or total nephrectomies^1,15^. Collected tissues were washed with cold HBSS (Invitrogen) and minced into ~ 1 mm cubes using sterile surgical scalpels followed by incubation at 37 °C for 2 h in Iscoves' Mod Dulbecco's Medium (IMDM) (Invitrogen) supplemented with 0.1% collagenase IV (Invitrogen). The processed tissue was then forced through 100 µm strainers to achieve a single cell suspension. After the digesting medium was removed, the cells were resuspended in a growth medium and plated in flasks. For passaging, cell detachment was performed via non-enzymatic cell dissociation solution (Sigma-Aldrich).

**Figure 1:**
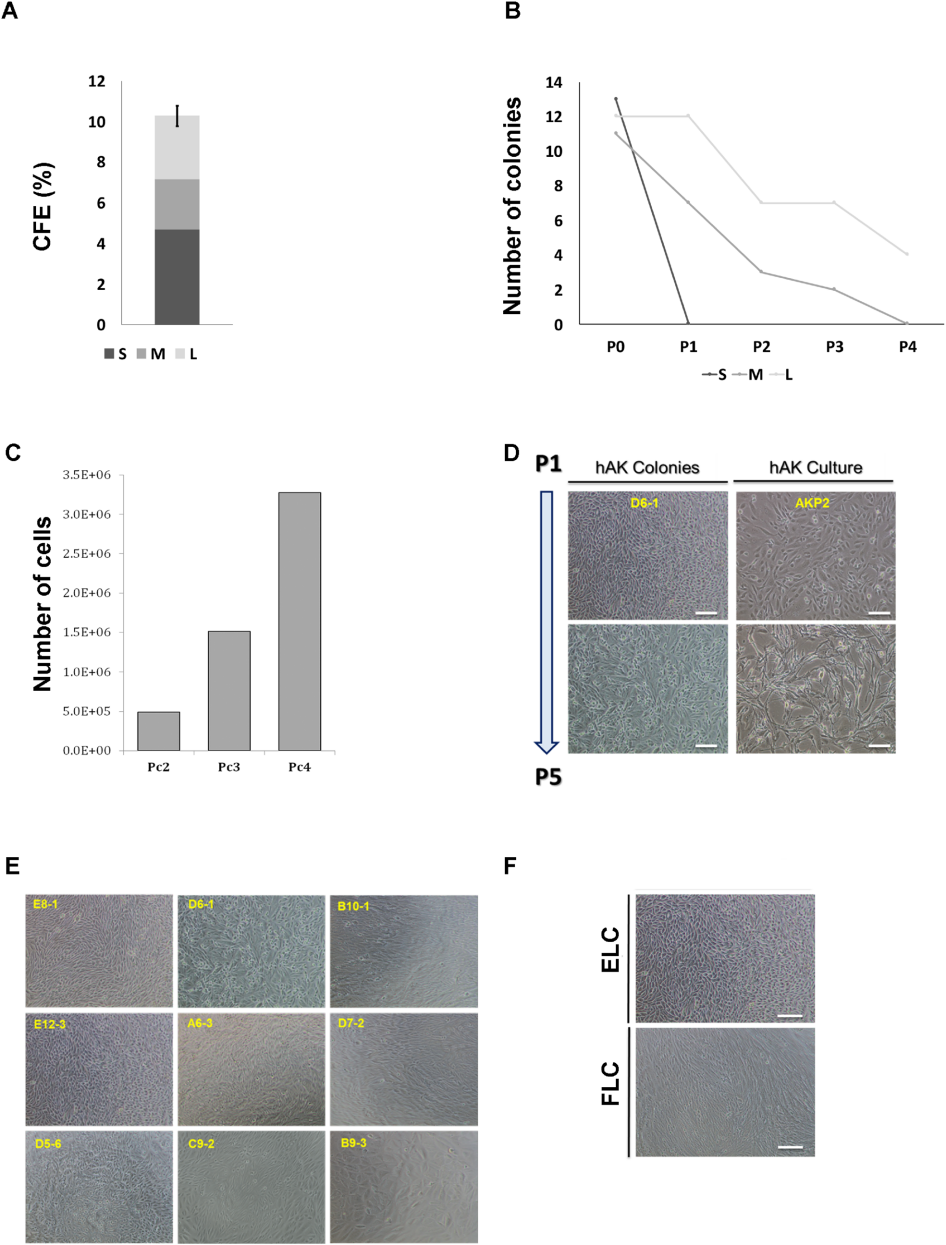
Establishment and characterization of single cell-derived colonies from human kidney. (**A**) CFE of single cell clones derived from fresh hAK cells according to their size (CFE% = 10.29 ± 1.1); Small (S)% = 4.69, Medium (M)% = 2.47, Large (L)% = 3.125; (**B**) Representative graph of self-renewal capacity of hAK-clones base on colony size. Data are presented for each passage as relative number of clones generated from the total number of cells plated. Most colonies surviving expansion were large (L) colonies (100% at P1 and 33.33% continued to expand for up to 4 passages) compared to medium (M) colonies that presented limited ability for clonal expansion (63% at P1 and none continued to expand beyond the 3rd passage) and small (S) colonies that failed to survive along passages altogether; (**C**) Representative graph of the expansion potential of hAK single cell derived clones. Clones originating from a single cultured AK cell were able to expand into approximately 3.4 * 10^6^ cells; (**D**) Representative morphology of an expanded hAK single cell derived clone (D6-1) along passages, compared to the heterogeneous hAK culture from which the clones was derived. A stable epithelial (EL) phenotype was preserved during clonal expansion for several month in contrast to the heterogeneous pool of cultured cells, which were already undergoing senescence and switching to a fibroblast-like morphology at the same time point. Scale bars, 100 μm; (**E**) Representative images of each of the 9 single cell clones at P0; (**F**) Representative photomicrographs of single cell clonal phenotypes. Two type of clones were generated: Epithelial-like (ELC) or Fibroblast-like (FLC). Scale bars, 100 μm.

Growth medium was comprised of two types of Serum-free media: 50% of (1) *N2 medium* (Biological Industries) supplemented with 1% Pen-strep 100 M, 1% L-glutamine, 0.4% B27 supplement (Gibco), 4 µg/ml heparin sodium (Intramed), 1% non-essential amino acids, 1% sodium pyruvate, 0.2% CD Lipid concentrate (all from Invitrogen), 2.4 mg/ml glucose, 0.4 mg/ml transferrin, 10 mg/ml insulin, 38.66 µg/ml putrescine, 0.04% sodium selentine, 12.6 µg/ml progesterone (all from Sigma-Aldrich), 10 ng/ml FGF and 20 ng/ml EGF and 50% of (2) *Conditioned Media* (CM) which was prepared by cultivating hFK cells in SFM for 48–72 h, using an inoculum density of 3.0 × 10^5^ cells mL^−1^. Spent medium was harvested by centrifugation, and the supernatant sterile filtered through a 0.22 μm bottle-top filter (Lab design) and kept at − 20 °C until used. Growth medium is referred to as conditioned SFM (CmSFM) hereafter. Cells were observed and photographed using Nikon Eclipse TS100 and Nikon Digital Sight camera (Fig. 1).

### Single cell cloning by limiting dilution assay and self-renewal assay

Cells were detached as described above and diluted with culturing medium. In order to achieve single cell per well, cells were plated onto pre-coated 96 well plates with Matrigel (BD Biosciences) at a density of 0.3 or 1 cell per with CmSFM as previously describe^16–18^. After 3–4 weeks the number of colonies was evaluated for each dilution. Clusters of cells were considered colonies when they were visible macroscopically. Colony forming efficiency (CFE) was determined from the formula CFE (%) = (number of colonies/number of cells seeded) × 100. Small colonies (S) = up to 10^4^ cells, Medium colonies (M) = 10^4^–10^5^ cells, Large colonies (L) = 10^5^ < cells. Upon confluence, single cell clones were detached using cell dissociation solution and re-plated in a larger fibronectin coated plate (Sigma-Aldrich) with CmSFM.

### Assessment of cell growth

4,000 cells were plated in triplicates and grown in 96-well plates over-night. The following day the medium was changed and supplemented with various concentrations of GW9662 (Sigma-Aldrich) or vehicle only (DMSO) as control, and the cells were further incubated for 48 or 96 h. Cell proliferation was measured using CellTiter 96 Aqueous One Solution Cell Proliferation Assay (Promega) according to the manufacturer's instructions. Briefly, the cells were incubated with the MTS solution for 3 h at 37 °C and absorbance at 492 nm was determined using the infinite F50 microplate reader (Tecan). Three independent experiments were carried out for each source.

### RNA sequencing and analysis

Bulk total RNA was quantified on an Agilent BioAnalyzer (Agilent Technologies) and aliquots of 270–500 ng were made into cDNA libraries using the TruSeq mRNA-Seq library kit (Illumina). Libraries were then sequenced 1 × 50 bases on the Illumina HiSeq 2500 platform.

Sequence data was analyzed using the protocol by Anders et al.^19^. Briefly, raw reads were aligned by TopHat2^20^ to the human hg19 genome. Aligned reads were counted by HTSeq^21^. Data normalization and differential gene expression was done by DESeq2^22^. Differentially expressed genes with *P* value ≤ 0.05 and absolute log2 fold change ≥ 0.5 were considered for downstream analysis. Euclidian distance and PCA analysis were performed on 1000 most variable genes. We used R packages ComplexHeatmap, and ggplot2 to generate the heatmaps and other plots in this study. Gene enrichment analysis was performed with GSEA^23^ we also performed gene set analysis using tstat value from DESeq2 with C5 GO gene sets from MSigDB.

The data were deposited at Gene Expression Omnibus (GEO, http://www.ncbi.nlm.nih.gov/geo/), accession number GSE144908.

### Statistical analysis

Error bars represent the mean ± SD, unless otherwise indicated. Statistical differences between cell populations were evaluated using the non-parametric, one sided sign test. Statistical differences between two group data were analyzed via Student's t test. For all statistical analysis, the level of significance was set as *p* < 0.05.

## Results

### Phenotypical analysis of single cell-derived colonies from the human adult kidney (hAK)

Human adult kidney cells derived from human nephrectomies were used for establishment of single cell clones. Cells were seeded in 96 well plates and cultured at a density of 0.3 cells per well^10^ and grown in defined CmSFM medium for 2 to 4 weeks (see Fig. S1 for schematic representation of experimental procedure). Single cell clones were initially characterized according to their size as large (L) medium (M) or small (S) clones. Colony Forming Efficiency (CFE)% was 10.94 ± 0.04 globally, S% = 4.69, M% = 2.47, L% = 3.125 (Fig. 1A). We next assessed in vitro self-renewal and expansion potential via serial passaging of the generated clones. Interestingly, most colonies surviving passaging and expansion were large at passage 0 (100% at P1 and 33.33% that continued to expand for up to 4 passages) compared to medium colonies that presented limited ability for clonal expansion (63% at P1, 27% at P2, 18% at P3 and none that continued to expand over to P4) and small colonies that failed to survive along passages altogether (Fig. 1B). Colonies originating from a single cultured AK cell demonstrated the potential to expand into up to 3.4E + 06 cells (Fig. 1C). Clones maintained their original morphology along passages for more than 3 months (Fig. 1D,E). A second subdivision of the single cell epithelial clones was done according to their morphology. Two distinct morphologic subgroups were observed; either cuboidal epithelial-like (EL) or spindle shaped fibroblast-like (FL) clones (Fig. 1F). Most EL clones (5 out of 6) were large and four were able to propagate in culture as opposed to only one large propagatable FL clone out of three.

Thus, single cell derived hAK clones can be phenotypically characterized according to clone size and morphology. Propagatable clones preserved their original phenotype during passages.

### RNA-sequencing analysis of human kidney clonal cultures

**Figure 2:**
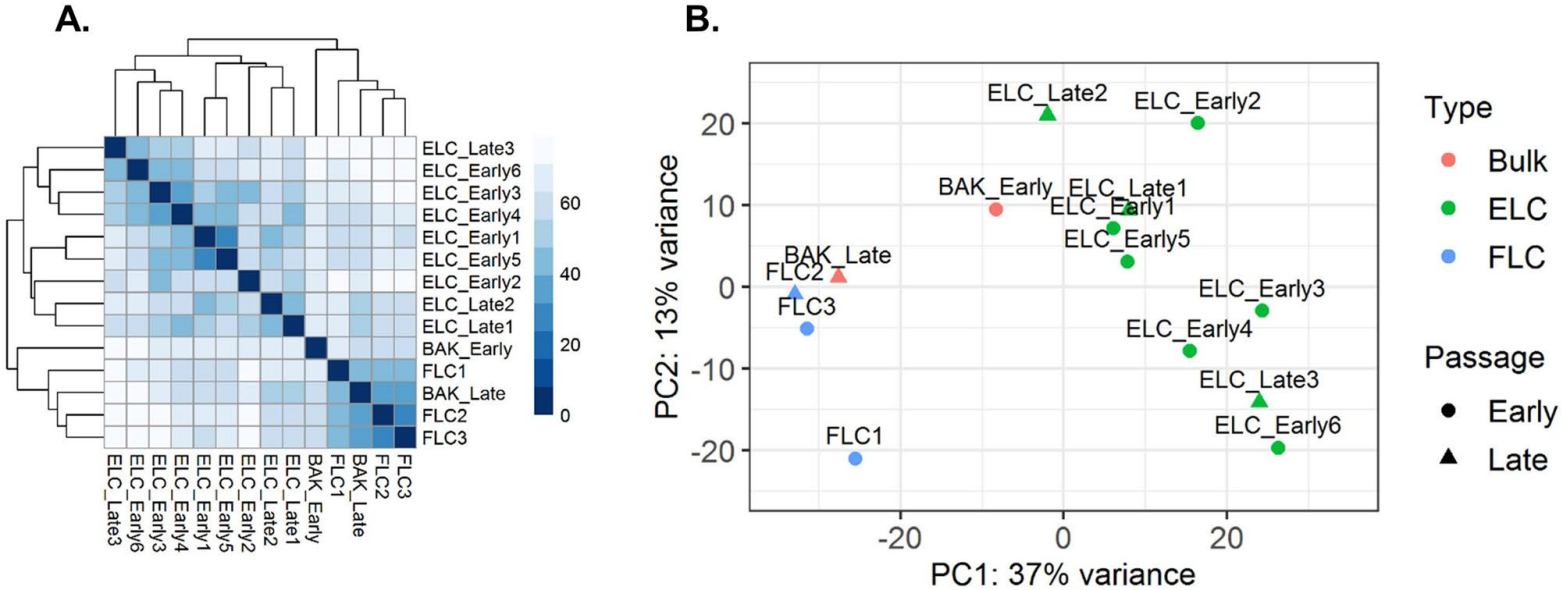
RNA-Sequencing analysis defines clone types upon in vitro expansion of human Adult Kidney single cell derived clones. (**A**) Euclidean distance analysis was performed on RNAseq data to illustrate variation in transcription levels between samples. FLCs are more similar to late BAK than to ELCs while early and late ELCs are clustered together. The distance between cell types is demonstrated by a heatmap. Darker color indicates lesser distance which means greater similarity; (**B**) Principal component analysis was performed on RNA-Seq data to illustrate variation in transcription levels between samples. Different colors denote culture type. Different shapes denote early or late passage. Bulk Human Adult Kidney-BAK; FLC-Fibroblast-Like Clones; ELC-Epithelial-Like Clones.

In order to characterize hAK clones at the molecular level we performed RNA-sequencing (RNA-Seq) of 9 hAK clones (Table S1) and bulk hAK cultures. Initial examination of EL, FL clones and bulk hAK cultures showed that early bulk hAK cultures (early hAK) undergo major transcriptional changes during propagation into late cultures (late hAK). Moreover, FL clones clustered with late hAK cultures, while all EL clones clustered together, distinct from the former, supporting a phenotypic-transcriptomic correlation and the maintenance of a stable transcriptome concomitant with the observed stable phenotype of the EL clones (Fig. 2A,B).

### Molecular characterization of clone types reveal sub-lineage restriction

**Figure 3:**
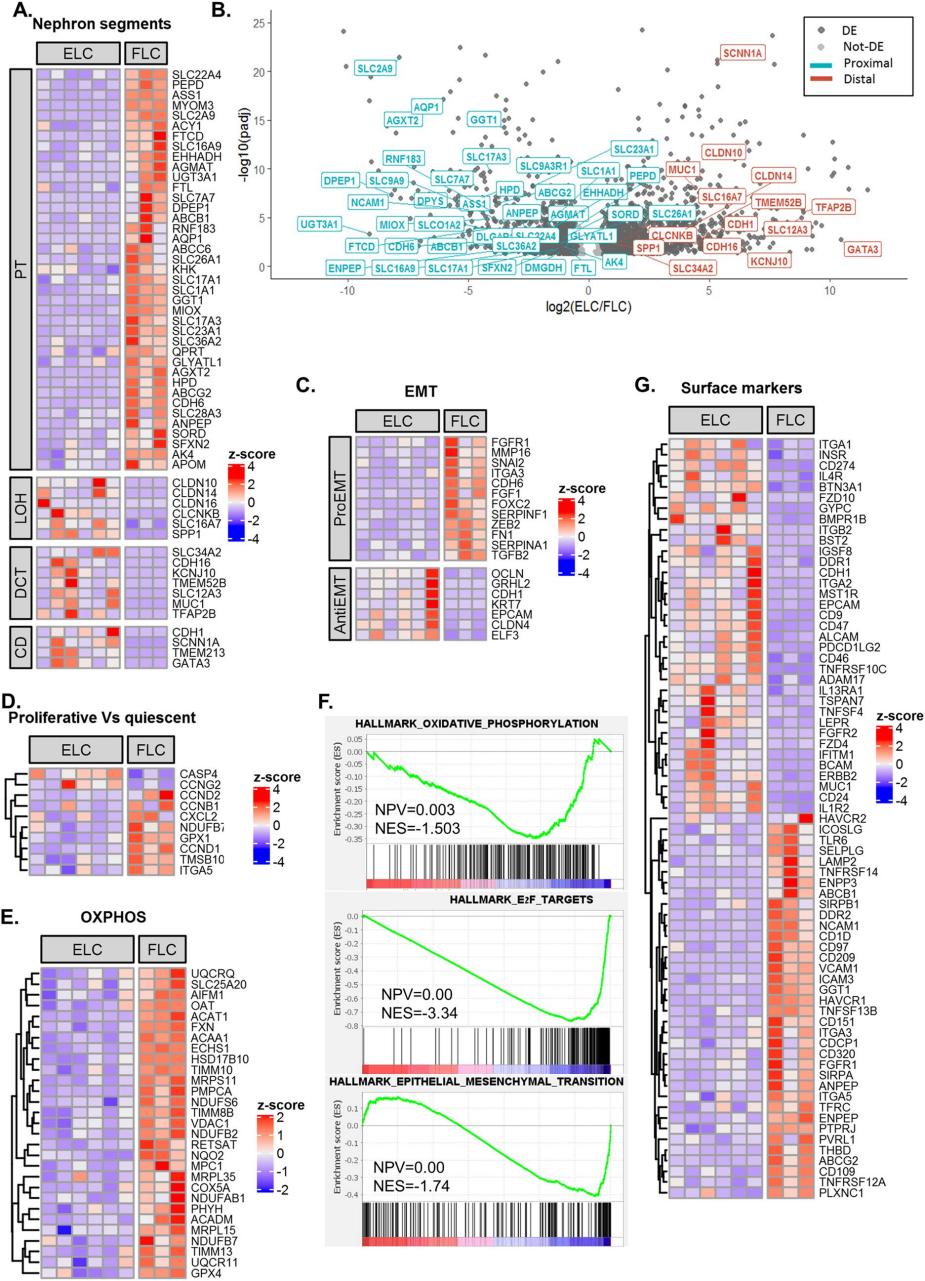
Molecular characterization of clone types reveal differences in the degree of epithelial maturation and nephron segment identity. (**A**) Heatmap representation of differentially expressed human nephron segment specific markers between FLC and ELC. FLC demonstrate a proximal nephron while ELC show distal nephron gene signature; (**B**) Volcano plot representation of all genes expressed in ELC and FLC with padj < 0.05. Dark-grey points denote differentially expressed genes between ELC and FLC (padj < 0.05, log2(fold-change) > 0.5). Kidney specific genes from different segments of the nephrons are labeled. While genes that mark the proximal segment of the nephron are down-regulated in ELC (cyan), genes that mark more distal segments (i.e. LOH, DCT and CD) of the nephron are up-regulated in ELC (red); (**C**–**D**) Heatmap representation of differentially expressed EMT (**C**), cell-cycle (**D**) and oxidative phosphorylation (**E**) genes between FLC and ELC, showing upregulation in FLC compared to ELC; (**F**) Gene set enrichment analysis (GSEA) showing enrichment of cell-cycle, EMT and oxidative phosphorylation genes in FLC; (**G**) Heatmap representation of differentially expressed surface markers between FLC and ELC; Abbreviations: ELC- Epithelial-like clones; FLC- Fibroblast-like clones; EMT- epithelial to mesenchymal transition; CD- cluster of differentiation.

In order to deepen our understanding of the driving forces preserving clonal epithelial cell growth, we sought to characterize the two morphologically distinct clone types (i.e. EL and FL) at the molecular level. Therefore, we queried genes that were differentially expressed between the two clone types (Table S2 summarizes DE genes). Analysis of known tissue-specific genes, which mark different nephron segments revealed the EL clones to possess a more distal tubular expression signature as manifested by elevation of SLC12A3, MUC1, CDH1 etc., while FL clones demonstrated a proximal tubular gene expression (i.e. high ENPEP, AQP1, ANPEP, CDH6, WT1, HAVCR1 etc.) (Fig. 3A,B; Table S2). To further support this conclusion we also tested nephron segment markers identified by single cell RNA-Seq analysis of human AK^24^, while genes identified as expressed in proximal clusters of the single cell analysis showed a decreased expression in EL clones in comparison to FL clones, genes related to more distal clusters were found to be elevated in EL clones, especially genes identified to be expressed by cells originated from LOH (Fig. S2A). While EL clones demonstrated preservation of epithelial gene expression including E-CAD/CDH1, GRHL2, EPCAM and KRT7 (Fig. 3C,F), FL clones showed elevated EMT genes including NCAM1, FGF1, SERPINA1, FN1, ZEB2. Genes up regulated in proliferative cells versus quiescent cells^25^ were found to be up-regulated in FL clones. (Fig. 3D,F). Additionally, oxidative phosphorylation genes were significantly higher in FL compared with EL clones (Fig. 3E,F). Concomitantly, gene ontology (GO) analysis revealed enrichment for mitochondrial related genes (i.e. electron transport, mitochondrial protein complex etc.) in line with mitochondrial abundancy in proximal tubular cells (Fig. S2B,C). Surface marker genes were also differentially expressed between EL and FL clones (Fig. 3G). Intriguingly, several Cluster of Differentiation (CD) coding genes with unique characteristics were also differentially expressed between the two clone types. These included, NCAM1 (CD56), a renal developmental marker which reactivates in the human adult kidney^14^ and that was highly expressed by FL clones. Additionally, CD109, a GPI-anchored protein that plays an impotent role in TGF beta signaling^26^, and CD209, an important innate immunity protein^27^, were up-regulated in FL clones. In contrast, CD24 and the immune response related CD coding genes (i.e. CD46, CD274) were highly expressed by EL clones. Moreover, EL clones expressed high levels of CD9, an exosome-specific marker. Intriguingly, CD133 was expressed by both clone types without a significant difference in expression levels between EL and FL clones.

As EL clones demonstrated preferable in vitro propagation capacity while preserving their phenotype for over 3 months, we next looked for possible "drivers" for clonal propagation. The most dominant signal transduction pathway in EL clones was the BMP/SMAD pathway, previously shown to take play a significant role in tubular regeneration as well as prevent fibrotic signaling in renal tubular cells following kidney injury^26^. Elevation of BMP ligands—BMP2, BMP4, BMP6 as well as BMP receptor—BMPR1 was observed in EL clones (Fig. S3). The inhibitory SMADs—SMAD7, SMAD6 are also upregulated in EL clones but even though they have an inhibitory effect on BMP pathway, they are also up-regulated by it^28^, putting the BMP pathway forward as a potential driver for human kidney epithelial clonal expansion. Thus, FL clones show EMT, cell-proliferation, oxidative phosphorylation genes while EL clones maintain epithelial markers. Interestingly, ccRCC is also characterized by EMT, cell cycle activation and oxidative phosphorylation^29–31^ indicating resemblance between ccRCC and FL clones. More specifically, the proximal markers SLC17A3 and VCAM1 that are both highly elevated in FL clones (Fig. S4B) were found to be upregulated in clear cell renal cell carcinoma (ccRCC)^24^. Other ccRCC markers such as ITGA5, GGT1, CDH6 and CA9 were also found to be upregulated in FL clones (Fig. S4B)^32^.

Finally, renal developmental genes may be activated in adult renal cells^33^, therefore we queried transcription factors (TFs) important for nephrogenesis in both clone types. EL clones showed expression of TFs expressing in ureteric bud (GATA3, HOXB7, HOXB9, GRHL2) and metanephric mesenchyme (MM) TFs (i.e. HOXD10, SALL2, PBX1) (Fig. S4A) . On the contrary, FL clones disclose MM and epithelial related progeny TFs (WT1, HNF1A, FOXC1) (Fig. S4A). Thus, EL clones may originate from both MM and UB, suggesting that distal tubule (MM-origin) clones may overlap with those of the collecting system (UB-origin) and cannot be fully discriminated.

### Molecular analysis of high-passage clones

In order to dissect the transcriptional processes taking place in EL clones during passages we also analyzed the transcriptome of late EL clones grown in culture for 3 months.

**Figure 4:**
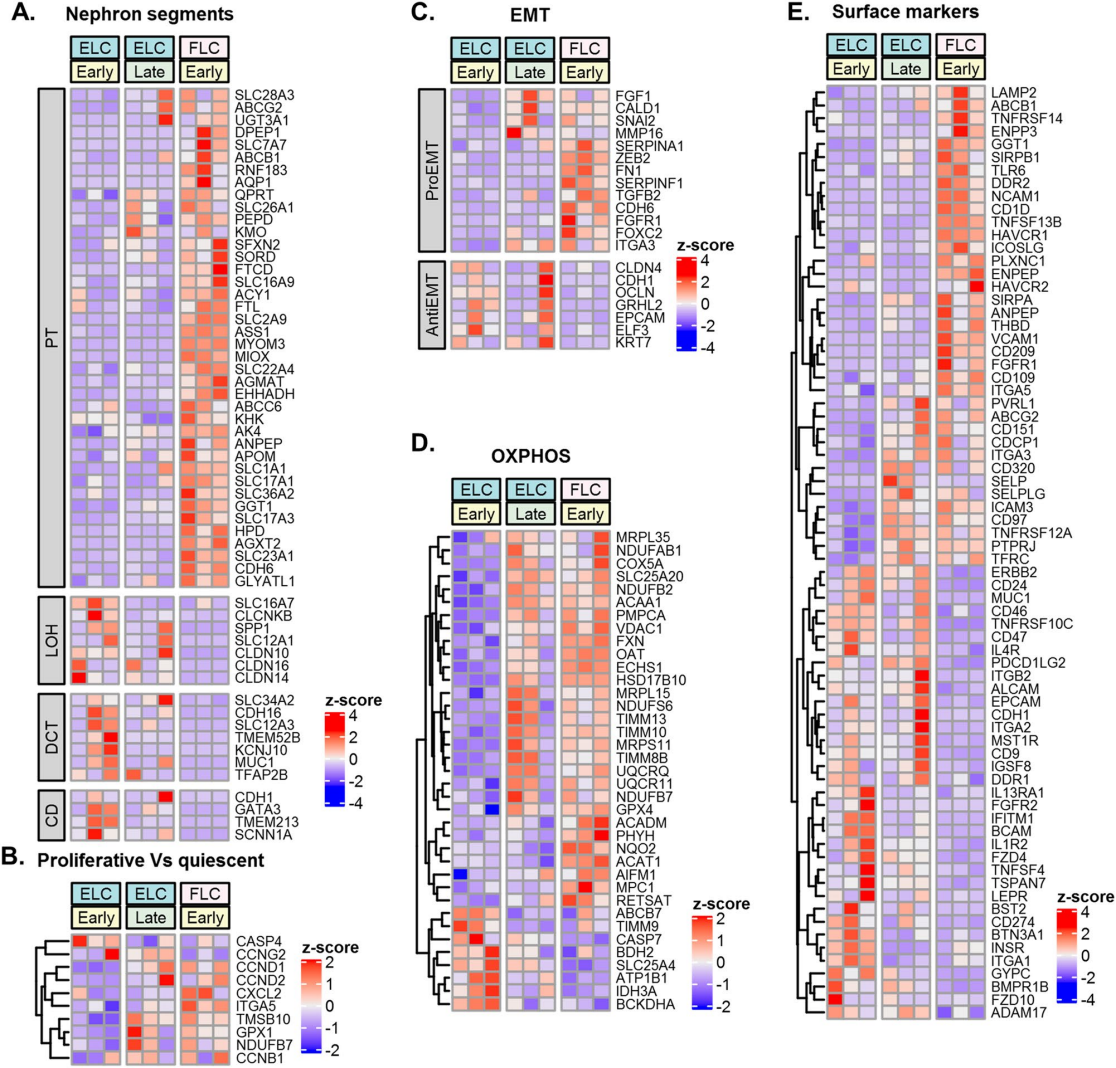
During propagation, EL-clones acquire FL-clonal transcriptional signature while preserving distal nephron segment identity. (**A**) Heatmap representations of differentially expressed genes between early ELC, late ELC and FLC. Along in vitro expansion, ELC demonstrate preservation of renal distal-segment identity; transcriptomic shift towards FL clonal signature in cell-cycle (**B**), EMT (**C**), and oxidative phosphorylation (**D**) genes; (**E**).Cluster of differentiation (CD) genes show preservation of CD9, CD46, CD24 among others alongside downregulation of CD274, FZD4, FZD10, BMPR1 and upregulation of CD151, CD97, CD320, during in vitro expansion of ELC clones Abbreviations: ELC- Epithelial-like clones; FLC– Fibroblast-like clones; EMT- epithelial to mesenchymal transition.

While late EL clones revealed preservation of renal distal-segment identity (Fig. 4A), a transcriptomic shift towards FL clonal signature was observed. This included an elevation in proliferation genes (i.e. CCND1. CCND2, CXCL2, etc.) (Fig. 4B) and epithelial to mesenchymal transition (EMT) genes (i.e. FGF1, CALD1, SNAI2, MMP16) (Fig. 4C). During in vitro propagation, EL clones display marked elevation of oxidative phosphorylation genes is in parallel to that observed in FL clones (Fig. 4D). Closer look at the expression of surface marker genes during in vitro propagation revealed preserved expression of CD9, CD46, CD24 among others. CD274, FZD4, FZD10, BMPR1 genes were downregulated and CD151, CD97, CD320 genes were upregulated. This expression pattern resembles that seen in early FL clones (Fig. 4E).

In conclusion, during in vitro propagation, EL clones preserve their original renal distal segment identity, while concomitantly acquiring characteristics of FL clonal transcriptional signature**.**

### Molecular characterization of early clonal versus bulk proliferation.

To further interrogate renal clonal behavior, we utilized Biomark comprised of simultaneous RQ-PCR analysis of 48 genes on early clonal growth by comparison to bulk cultures. We queried 12 clones and compared them to 6 bulk counterparts. Characteristically early clonal proliferation was manifested by reduced MKI67 and TOP2A (Fig. S5A), reduced overall renal marker identity and elevated renal developmental molecules (PAX2, SALL1, CDH6, CITED1, ALDH1A1; Fig. S5A). Interestingly, WNT5A was activated in early clonal growth. Finally, although overall decreased expression of renal identity markers in early clones was noted compared to bulk cultures, we could detect an expression of multiple nephron segment markers in clones (Fig. S5B). RNA seq comparison of clones to bulk culture confirmed renal developmental gene activation (PAX2/SALL1) with MKI67 reduction (Fig. S5C,D).

## Discussion

Previous studies have highlighted in vivo segment-specific clonal proliferation of tubular epithelial cells, as a driver for cell replacement in the adult kidney^26^. Herein, we have calibrated a culture system that allows ex-vivo single cell derived clonal growth of the human kidney in order to delineate its phenotypic and molecular characteristics by transcriptomics. Our study shows that the kidney contains cells capable of generating clones with different phenotypes and proliferative capacity. We demonstrate that some clones not only proliferate, but also maintain a stable phenotype and quiescence-proliferation balance as manifested by constant replication time, over consecutive passages indicating the unique potential of the clonal assay. Remarkably, human kidney cultures are endowed with clonogenic self-renewal enabling clonal growth over 3 months and clonal output of up to 3.4 * 10^6^ differentiated renal cells that eventually far exceeds the in vivo mouse clonal output.

A comparison of clonal proliferation showed restriction to a renal sub-lineage. Phenotypically, we observed cultures with a more mesenchymal appearance, initial proliferation burst, generating clones of variable size with a reduced propagation capacity over passages. Additional phenotype demonstrated cuboidal epithelial morphology, steady proliferation, formation of larger clones and propagation over several passages in culture. This clonal heterogeneity was resolved by RNA sequencing when comparing the two clone types; FL type clones represent clones originating from the proximal tubules as manifested by high expression of proximal tubule markers (i.e. ENPEP, AQP1, CD13, SLC17A3 among others) (see Fig. 3). A comparison of the FL signature with data of Young et al. of single cell RNA-Seq analysis of adult human kidney^22^ pin pointed PT1/PT2 and not PT3 as cell of origin (Fig. S2A) In contrast, EL clones derive from distal renal segments displaying overexpression of distal tubule markers (i.e. SLC12A3, MUC1, CDH1 among others). This clear separation ignited a more in-depth molecular analysis of clone types. Concomitant with a proximal tubule transcriptional signature, FL clones demonstrated enhanced EMT, cell cycle and embryonic stem cell gene expression, suggestive of concomitant proliferative and de-differentiation states. Interestingly, the regenerative response to acute kidney injury is dependent on proximal tubular cells undergoing concurrent dedifferentiation and proliferation^7,8^. Thus, transcriptional signature of FL clones mimics the regenerative response of proximal tubular cells to an acute kidney insult. In this regard, FL clones may inform on the clonal in vivo regenerative response; As such it is noteworthy to mention NCAM1 (CD56), previously indicated as a marker activated in a subset of cultured human adult kidney tubular cells with clonogenic properties and in Wilms’ tumor stem cells ^34–36^ and here indeed shown to be a molecular marker of proximal clonal proliferation alongside HAVCR1/KIM1. Other markers included CD109, a GPI-anchored cell surface glycoprotein upregulated in cancer^37,38^. CD109 is a member of the TGF beta signal transduction, negatively regulating TGF beta/SMAD complex in keratinocytes^39^, and has been recently put forward as a marker for cancer stem-like cells in epithelioid sarcoma^40^. Epithelioid sarcoma are rare malignant tumors of mesenchymal origin that histologically display mix epithelial and mesenchymal traits^41^. Thus, expression of CD109 may illustrate the more mesenchymal state of FL clones derived from a single kidney epithelial cell. Intriguingly, the gene signature set of proximal clonal proliferation apparent in FL clones shared multiple molecular markers identified in single cell RNA-Seq of clear cell RCC of which SLC17A3 and VCAM1 were most notable. These molecules were overexpressed in FL clones along with ITGA5, GGT1, CDH6 and CA9 all previously shown to be highly expressed by clear cell RCC^24,42–45^. Thus, intriguingly proximal clonal proliferation and behavior may be viewed as precursor cell lesion for RCC^46^.

Oxidative phosphorylation, respiratory chain, mitochondrial matrix and envelope genes were also significantly elevated in FL clones. Thus, clones do not appear to switch to aerobic glycolysis (Warburg effect^47^) as expected for highly proliferating cells, but rather increase oxidative phosphorylation. This observation is in line with inherent mitochondrial abundancy of proximal tubule cells, relying on aerobic respiration in order to be able to account for their high metabolic demands (reabsorption of 80% of the glomerular filtrate), whereas distal parts of the nephron possess a greater capacity to shift from oxidative phosphorylation to glycolysis^48^. In addition, increased oxidative phosphorylation was recently put forward as harboring a significant role in cell proliferation through a process of mitochondrial fusion^49^.

In contrast, distal tubule transcriptional characteristics of the EL clones were accompanied by elevated expression of epithelial differentiation markers (i.e. CDH1, EPCAM, KRT7, GRLH2 etc.) alongside relatively decreased expression of EMT and cell cycle genes. Since more quiescent EL clones possess increased in vitro propagation capacity compared to FL clones, it seems that the degree of epithelial differentiation is crucial for clonal propagation. High CD24 expression coincides with the expression of epithelial differentiation markers in EL clones and as such may represent a marker associated with epithelial-type clonal proliferation. Interestingly, Elevation of CD genes related to immune response and exosomal communication was noted in EL clones. The former included CD46 associated with compliment pathway retardation^50^ and CD274, linked with inhibition of T cell function, all three were shown to decrease kidney allograft rejection^51,52^. CD9, an exosome-complex gene was also elevated in EL clones. It was shown to take part in exosomal communication between the proximal and distal tubular segments in the human kidney, resulting in decreased production of reactive oxygen species (ROS) in distal tubular cells^53^. Thus, alongside distal tubular cells being inherently more resistant to ischemia as well as to oxidative stress^54^, this surface marker expression pattern suggest a possible protective role against oxidative stress during epithelial-type clonal growth.

During EL clonal growth, BMP signaling was the most prominent pathway activated (i.e. high BMPR1, BMP2, BMP4, BMP6, SMAD1, SMAD6 expression) (Figs. 3 and S3). BMP signaling is involved in nephron patterning during kidney development and later on, operates selectively along specific nephron segments^55^. In accordance with this pathway activation in EL clones that maintain both epithelial phenotype and epithelial transcriptional signature, several BMP proteins (i.e. BMPR1, BMP2, BMP4, BMP6, BMP7 etc.) were shown to possess an anti-fibrotic effect following acute kidney injury^56–58^. Moreover, activation of the BMP-SMAD pathway is seen in epithelial cells that are re-lining the nephron following acute injury^57^. Additionally, in a model of interstitial nephritis, induced inactivation of the ALK3 receptor (a high-affinity receptor for BMP2 and BMP4) sensitizes proximal tubule cells to injury and promotes fibrosis^57^. Thus, taken together, activation of the BMP signaling pathway EL clones corresponds to its role in renal epithelialization and epithelial maintenance as well as its anti-fibrotic effects. On the other hand, downregulation of this pathway in FL clones is in accordance with the pro-fibrotic response of proximal tubule cells to injury in its absence.

Importantly, a consecutive analysis of transcriptional changes during EL clonal propagation revealed preservation of segment identity while concomitantly exhibiting a transcriptional shift towards some aspects of the FL-clonal signature set. These changes were manifested as elevation in EMT and cell cycle genes alongside a metabolic shift towards oxidative phosphorylation and occurred although the EL-epithelial cell phenotype was still apparent in culture. Hence, it remains to be determined whether a continued BMP signal and molecules that intervene with mitochondrial function can support prolonged clonal epithelial growth.

While comparison between clone types revealed proximal/distal segmentation, we also performed a comparison between early clonal proliferation and bulk proliferation. This comparison unraveled that early clones are relatively more quiescent (reduce Ki-67) than bulk counterparts, overexpress some renal developmental genes, mostly CDH6, PAX2 and SALL1 and activate WNT5A. This clonal state is associated with an overall relative reduction in renal identity markers that specify nephron segments. Interestingly, although a reduction is noted, the expression of multiple nephron segments (both proximal and distal markers) and a clonal cell state encompassing multiple identities could be recognized in early clones, similarly to what was find previously suggested by Schutgens et al. and Rudman-Melnick et al.^59,60^. Importantly, culture conditions might have influenced these findings representing a potential limitation of this work. Accordingly the development of a transient progenitor-state of mature renal cells at the very onset of clonal growth can potentially be clarified in vivo by single cell RNA-Seq of specific kidney segments and epithelial cell states in steady state and during a regenerative process.

To conclude, we show that the lineage-restricted precursor governs single cell clonal growth ex vivo, sharing similarities to in vivo clonal behavior^2^. Interestingly, in vitro, the kidney segment and the level of epithelial differentiation dictate clonal growth and propagation over time. These data suggest that for reconstruction of varying renal lineages for human adult kidney based organoid technology and kidney regeneration ex-vivo use or aggregation of multiple heterogeneous precursors are warranted.

## Supplementary information

Supplementary Information 1.

Supplementary Information 2.

Supplementary Information 3.

## Data Availability

The datasets generated during and/or analysed during the current study are available in the Gene Expression Omnibus (GEO) repository [http://www.ncbi.nlm.nih.gov/geo/]. Accession number GSE144908.
