# Author Correction: Human kidney clonal proliferation disclose lineage-restricted precursor characteristics

**DOI:** 10.1038/s41598-021-86157-7

**Published:** 2021-03-22

**Authors:** Osnat Cohen-Zontag, Rotem Gershon, Orit Harari-Steinberg, Itamar Kanter, Dorit Omer, Oren Pleniceanu, Gal Tam, Sarit Oriel, Herzl Ben-Hur, Guy Katz, Zohar Dotan, Tomer Kalisky, Benjamin Dekel, Naomi Pode-Shakked

**Affiliations:** 1 Pediatric Stem Cell Research Institute, Edmond and Lily Safra Children’s Hospital, Sheba Medical Center, Tel-Hashomer, Israel; 2 Department of Urology, Sheba Medical Center, Tel-Hashomer, Israel; 3 The Talpiot Medical Leadership Program, Sheba Medical Center, Tel-Hashomer, Israel; 4 Faculty of Engineering and Bar-Ilan Institute of Nanotechnology and Advanced Materials (BINA), Bar-Ilan University, Ramat Gan, Israel; 5 The Joseph Buchman Gynecology and Maternity Center, Sheba Medical Center, Tel-Hashomer, Israel; 6 Division of Pediatric Nephrology, Edmond and Lily Safra Children’s Hospital, Sheba Medical Center, Tel-Hashomer, Israel; 7 Sackler Faculty of Medicine, Tel-Aviv University, Tel-Aviv, Israel; 8 L.E.M. Laboratory of Early Detection, Nes Ziona, Israel; 9 Department of Obstetrics and Gynecology, Shamir Medical Center, Be’er Ya’akov, Israel

Correction to: *Scientific Reports* 10.1038/s41598-020-78366-3, Published online 16 December 2020

The original version of this Article contained errors in the spelling of the authors Osnat Cohen-Zontag, Rotem Gershon, Orit Harari-Steinberg, Itamar Kanter, Dorit Omer, Oren Pleniceanu, Gal Tam, Sarit Oriel, Herzl Ben-Hur, Guy Katz, Zohar Dotan, Tomer Kalisky, Benjamin Dekel, and Naomi Pode-Shakked, which were incorrectly given as Cohen-Zontag Osnat, Gershon Rotem, Harari-Steinberg Orit, Kanter Itamar, Omer Dorit, Pleniceanu Oren, Tam Gal, Oriel Sarit, Ben Hur Herzel, Katz Guy, Zohar Dotan, Kalisky Tomer, Dekel Benjamin, and Pode-Shakked Naomi.

These errors have now been corrected in the PDF and HTML versions of the Article, and in the accompanying Supplementary Information file.

