# Supplementary material for: Human kidney clonal proliferation disclose lineage-restricted precursor characteristics
Source: Sci Rep. 2020 Dec 16;10:22097. doi: 10.1038/s41598-020-78366-3 (PMC7745030; doi:10.1038/s41598-020-78366-3)
Supplement: Supplementary file 1 — Supplementary Information 1. [file 41598_2020_78366_MOESM1_ESM.docx]

**Human kidney clonal proliferation disclose lineage-restricted precursor characteristics**

Osnat Cohen-Zontag^1,7,*^, Rotem Gershon^1,7,*^, Orit Harari-Steinberg^1,7,*^, Itamar Kanter^4^, Dorit Omer^1,7^ , Oren Pleniceanu^1,7^, Gal Tam^4^, Sarit Oriel^4^, Herzl Ben-Hur^8,9^, Guy Katz^1,3,5,7^, Zohar Dotan^2,7^, Tomer Kalisky^4,#^, Benjamin Dekel^1,6,7,#,^^, Naomi Pode-Shakked^1,3,7,#^.

^1^Pediatric Stem Cell Research Institute, Edmond and Lily Safra Children's Hospital, Sheba Medical Center, Tel-Hashomer, Israel

^2^Dept of Urology, Sheba Medical Center, Tel-Hashomer, Israel

^3^The Talpiot Medical Leadership Program, Sheba Medical Center, Tel-Hashomer, Israel

^4^Faculty of Engineering and Bar-Ilan Institute of Nanotechnology and Advanced Materials (BINA), Bar-Ilan University, Ramat Gan, Israel.

^5^The Joseph Buchman Gynecology and Maternity Center, Sheba Medical Center, Tel-

Hashomer, Israel

^6^Division of Pediatric Nephrology, Edmond and Lily Safra Children's Hospital,

Sheba Medical Center, Tel-Hashomer, Israel

^7^Sackler Faculty of Medicine, Tel-Aviv University, Tel-Aviv, Israel

^8^L.E.M. Laboratory of Early Detection, Nes Ziona, Israel

^9^Department of Obstetrics and Gynecology, Shamin Medical Center, Israel

^*^These first authors contributed equally to this work

^#^These senior authors contributed equally to this work

^^^Correspondence:

Benjamin Dekel MD, PhD 
Pediatric Stem Cell Research Institute
Edmond & Lily Safra Children's Hospital, 
Sheba Medical Center 
E-mails: [binyamin.dekel@sheba.health.gov.il](mailto:binyamin.dekel@sheba.health.gov.il) or [benjamin.dekel@gmail.com](mailto:benjamin.dekel@gmail.com)

# Supplemental information – Table of content:

Supplemental figures and legends 3

Figure S1| 3

Figure S2| 4

Figure S3| 6

Figure S4| 7

Figure S5| 8

legend for Supplemental tables 9

Table S1| 9

Table S2| 9

Table S3| 9

##
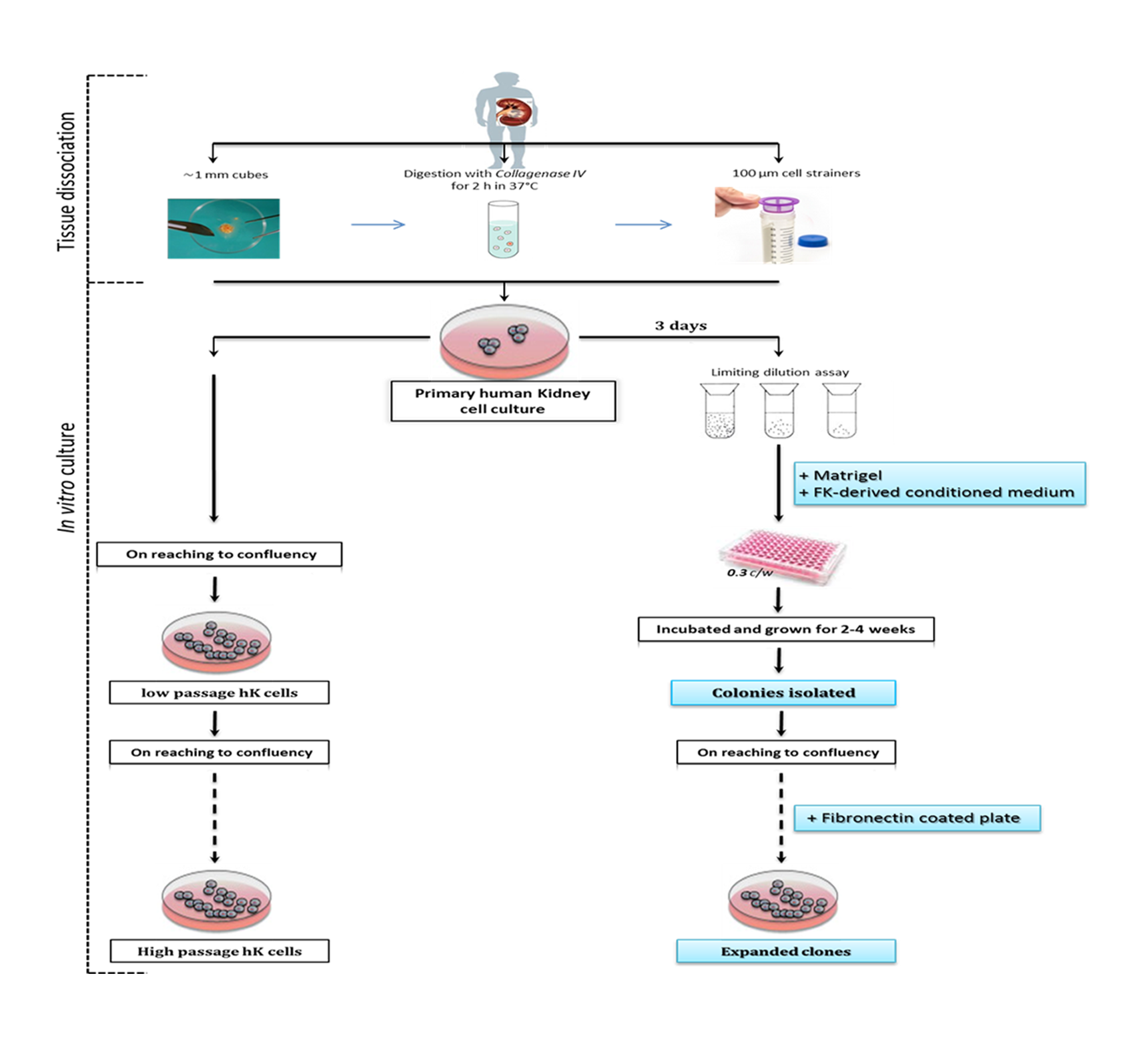
Supplemental figures and legends

###

### Figure S1| Schematic representation of the method used for establishment of an efficient and reliable method for forming single cell clones from primary human

### kidney. Flow diagram of the steps involved in adult kidney clones isolation and expansion.

###

###
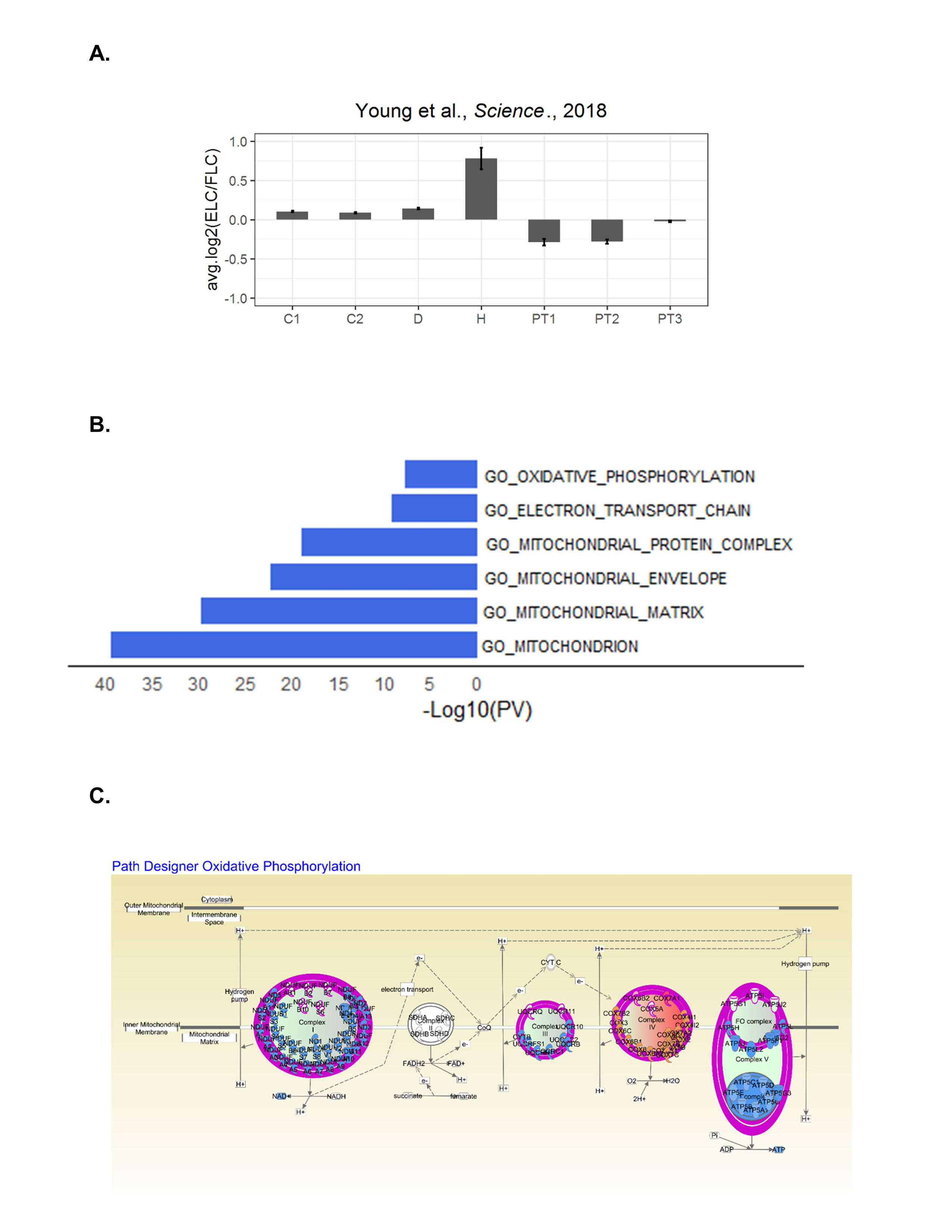


### Figure S2| (A) Bar plots represent average value of log2 ratio between ELC and FLC of genes uniquely expressed at different nephron segments identified by single cell RNA analysis [24]. Genes expressed in proximal clusters showed a decreased expression in EL clones in comparison to FL clones, while genes of more distal clusters are elevated in EL clones. (B) Gene set enrichment analysis of differentially expressed statistics comparing ELC to FLC reveal an enrichment of mitochondrial-related GO terms in genes down-regulated in ELC ); (C) Schematic representation of changes in mitochondrial related genes in FLC and ELC showing most complexes to be highly expressed by FLC. Annotations: Abbreviations: ELC- Epithelial-like clones, FLC- Fibroblast-like clones.

###
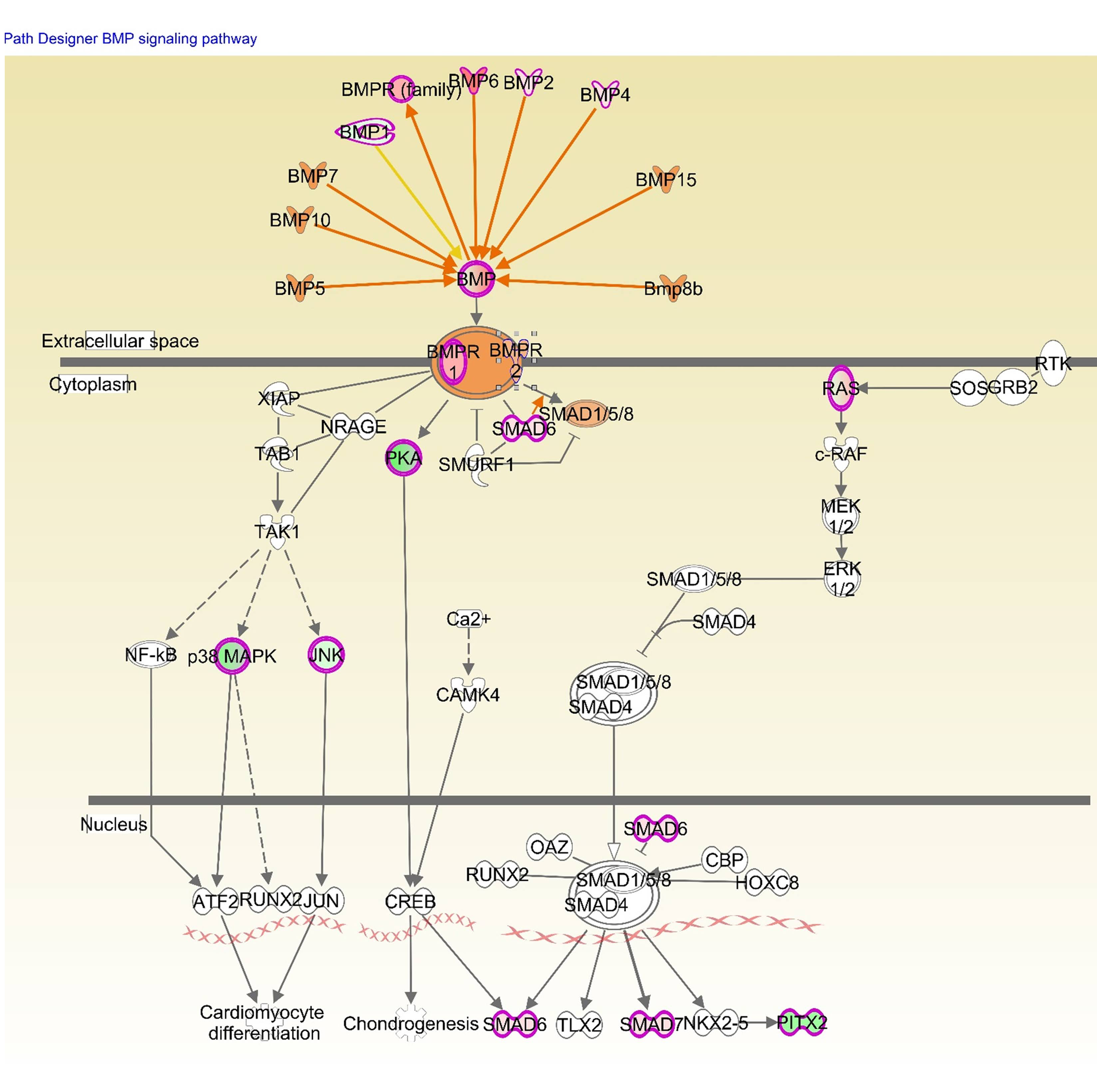


###

### Figure S3| Schematic representation of changes in BMP/SMAD signal transduction pathway genes, showing many of its members to be highly expressed in ELC. Abbreviations: ELC-Epithelial-like clones.


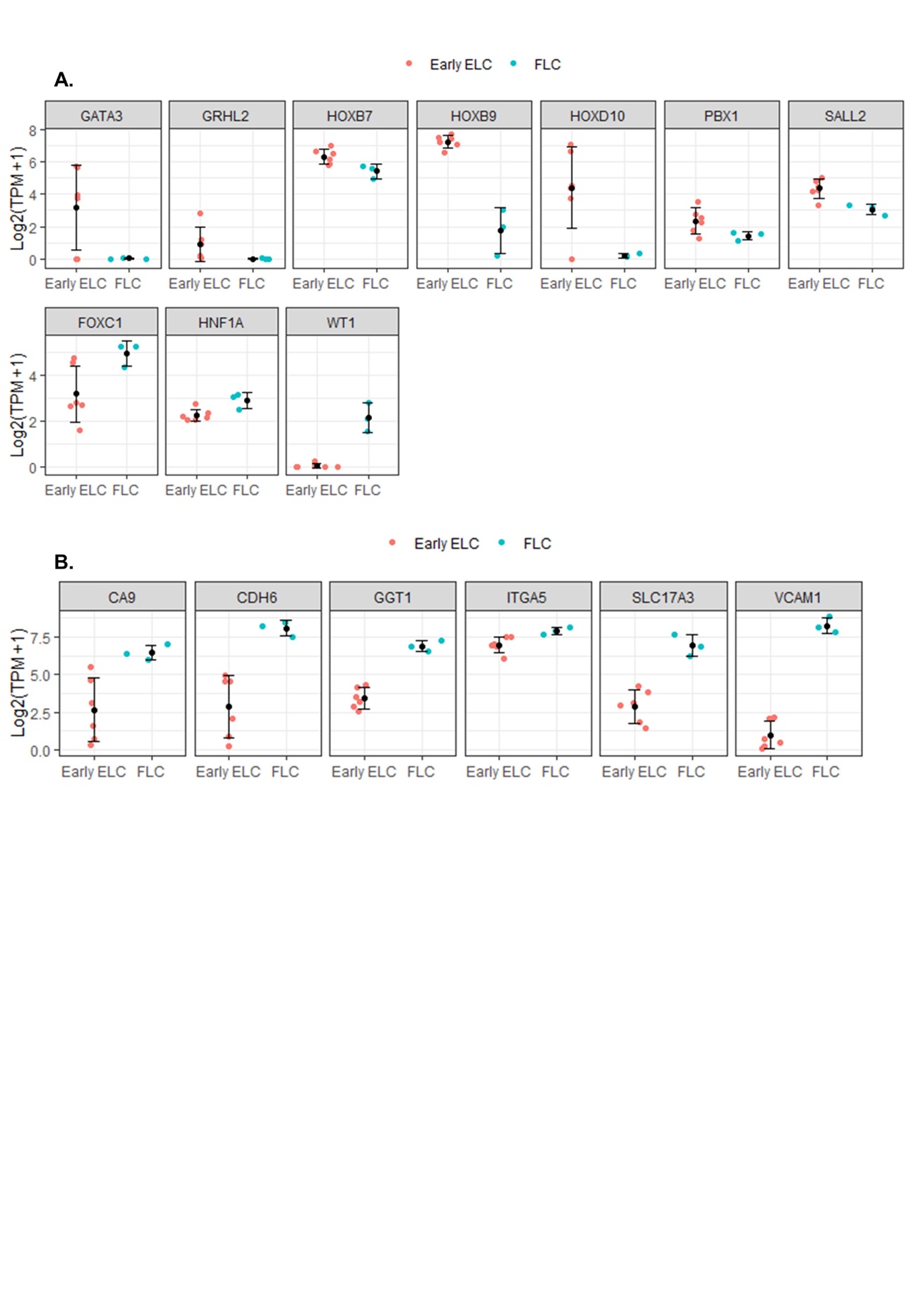


### Figure S4| (A) Expression levels of nephrogenesis transcription factors differentially expressed between Early EL clones (red) and FL clones (turquoise). While genes upregulated in ELC are expressed in both the UB and the MM during renal development, genes upregulated in FLC are known to be expressed only in the MM; (B) Expression levels of Renal Cell Carcinoma (RCC) markers differentially expressed between Early EL clones (red) and FL clones (turquoise). Genes upregulated in FLC are known to be expressed in RCC. Gene expression levels were identified via RNA-Seq.

###
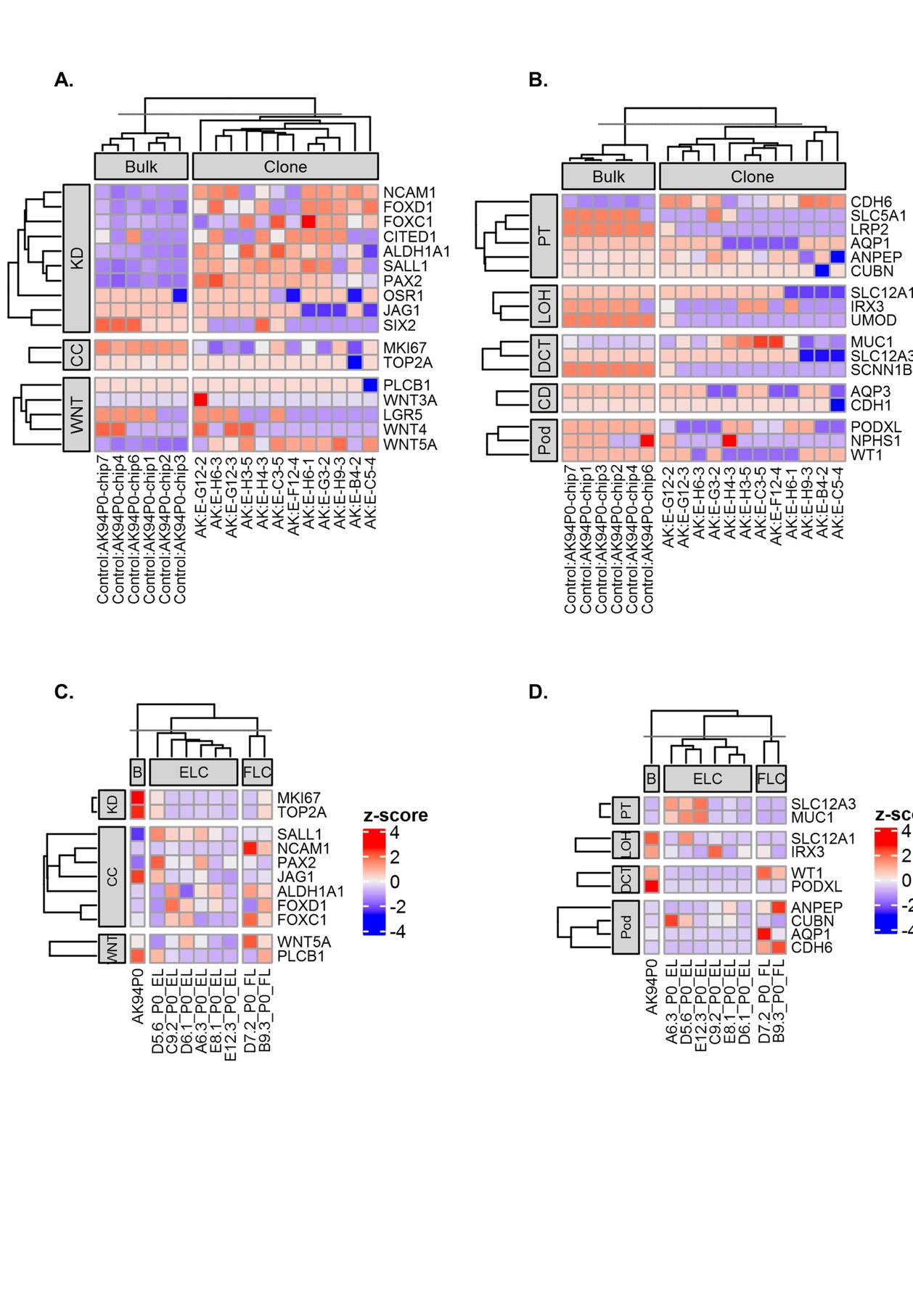


### Figure S5| Molecular characterization of early clones versus bulk proliferation via biomark and RNA-Seq analysis. (A) Heatmap represents expression pattern of kidney development (KD), cell cycle (CC) and wnt signaling (WNT) genes in Bulk human adult kidney cultures and early clones revealed by biomark analysis; (B) same as A for nephron segment specific markers genes; (C-D) same as A-B but expression pattern revealed via RNA-Seq analysis.

## Legend for Supplemental table

### Table S1 | Description of the characteristics of each of the 9 adult kidney single cell-derived clones on which the RNA sequencing analysis was performed.

| Name | Morphology | Colony Size | Clonogenic Expansion |
| --- | --- | --- | --- |
| E8-1 | Early EL | L | P3 |
| D5-6 | Early EL | L | P1 |
| E12-3 | Early EL | M | P1 |
| D6-1 | Late EL | L | P4 |
| C9-2 | Late EL | L | P3 |
| A6-3 | Late EL | M | P4 |
| D7-2 | FL | L | P1 |
| B10-1 | FL | L | P4 |
| B9-3 | FL | S | P0 |

### Table S2 | Differentially expressed (DE) genes between EL and FL clones. Genes were divide to groups: up-regulated in EL clones compared to FL clones and down-regulated in EL clones compared to FL clones.

Uploaded as a separated excel file.

### Table S3 | Full RNA-Seq Data row counts used for the expression analysis of the different cultures (AK, ELC, FLC) generated from Early or Late passages.

Uploaded as a separated excel file.
